# A vector-agent approach to (spatiotemporal) movement modelling and reasoning

**DOI:** 10.1038/s41598-022-22056-9

**Published:** 2022-12-07

**Authors:** Saeed Rahimi, Antoni B. Moore, Peter A. Whigham

**Affiliations:** 1 School of Surveying, University of Otago, 310 Castle Street, Dunedin, 9013 Otago New Zealand; 2 Department of Information Science, University of Otago, 60 Clyde Street, Dunedin, 9013 Otago New Zealand

**Keywords:** Computational science, Computer science

## Abstract

Modelling a complex system of autonomous individuals moving through space and time essentially entails understanding the (heterogeneous) spatiotemporal context, interactions with other individuals, their internal states and making any underlying causal interrelationships explicit, a task for which agents (including vector-agents) are specifically well-suited. Building on a conceptual model of agent space–time and reasoning behaviour, a design guideline for an implemented vector-agent model is presented. The movement of football players was chosen as it is appropriately constrained in space, time and individual actions. Sensitivity-variability analysis was applied to measure the performance of different configurations of system components on the emergent movement patterns. The model output varied more when the condition of the contextual actors (players’ role-areas) was manipulated. The current study shows how agent-based modelling can contribute to our understanding of movement and how causally relevant evidence can be produced, illustrated through a spatiotemporally constrained football case-study.

## Introduction

Studying dynamic geographic phenomena, relationships, and processes are major activities in spatially-based sciences^1^. The key to pursue these activities is the representation of space and time at both observation- and model-level. Despite progress in data models^2–10^, process-modelling remains an open task within GIScience and GeoComputation related studies. This is due to the inherent complexity of such processes that often involve individuals’ decisions, driven by their capabilities, objectives, and perceptions of the environment.

Autonomous movements, for example, are dynamic geographic processes driven by goal-oriented autonomous entities who decide why and how to move based on their internal state, motion and navigation capacities, as well as external factors^11^. Movement behaviours are accordingly complex, subject to a multi-layered decision-making process that itself is subject to temporarily dynamic micro-level causal connections of compound and spatially distributed entities. Accordingly, the potential and challenges of exploring movement behaviours have recently attracted both theoretical and applied interest in many GIScience-related studies^12–15^.

### Proposing agents for movement modelling

Implementing agents has recently become a promising approach to process modelling in various fields. Agent-based Models (ABMs) are a computational representation of some aspect of the real world through the actions of a collection of adaptive decision-makers (agents) which are driven by a set of rules governing interaction with themselves and their environment^16^. They are increasingly being accepted as a powerful tool that allows for the explicit representation of the processes underlying large-scale animal movement patterns^17–21^. They are also popular in pedestrian modelling^22–26^, in studying the effects of movement behaviours on epidemic spread^27,28^, and in crowd movement simulation^29,30^. One recently introduced and growing discipline for examining the physical aspects of autonomous moving agents is Active Matter, particularly in living and engineering systems (see^31^ for a comprehensive introduction).

ABMs are now well-established in GIScience^32–35^. A decade after introducing Cellular Automata^36^, fully-fledged ABMs were implemented in the geographical sciences^33^. Not long after, researchers started to define a specific class of autonomous spatial agents that are identifiable, georeferenced, have characteristics with some level of spatial extension^37^. ‘Geographic Automata’^38^ and ‘GeoAgents’^39^ are such spatial agents. These agents are designed to reason spatially, meaning that their actions are geographically/geometrically dependent, while interacting with an explicitly scale-dependent geographic environment^39^. They are usually represented as spatial features (i.e. points, lines or polygons). Such characteristics led to the concept of ‘vector-agents’^40^, whose actions are expected to change the geometric attributes (e.g., position, shape, and size) of themselves, other objects or agents. Vector-agents have been adapted and implemented in a range of GIScience related studies, including urban studies^41^, land use modelling^42^, and remote sensing^43,44^.

Vector-agents, empowered by object-oriented data models, facilitate process models to represent space and time alongside individual behaviours. The following characteristics suggest that the agent-based approach, more specifically the vector-agent paradigm, is an appropriate method to represent possible movement behaviours under different causal assumptions that ultimately lead to explaining their underlying mechanisms:

- Agents in ABMs are usually designed with different characteristics, desires, moving capability, learning capacity, and interaction strategies.
- They are often programmed with different mental models and perceptions of the environment within which they move and interact.
- Agents can represent autonomy, as they can be encoded to determine their goals and choose among alternative actions to achieve them.
- Agents are often situated within space and time. Modellers can specify various environments and contextual constraints.
- They change the environment with their actions and are influenced by this environment that is being shaped by themselves and others.
- Specific to the vector-agents, they can effectively represent the dynamic behaviour of moving objects at the fine level of spatiotemporal resolution.

However, movement studies still appear to ignore some aspects of Agent-based Modelling (ABM). First, most models tend to utilize ABM for representing the dynamics of spatial phenomena rather than for its ability to clarify the underlying mechanisms of movement processes. More importantly, when there is a component of the causes and consequences of movement, it tends to be at the aggregated level rather than individual level. For example, Ahearn et al.^45^ have modelled the behaviour of tigers and their interactions with wild and domestic prey. Although their model is mainly based on the implicit movement behaviour of tigers, its outcome is limited to the number of tigers (born, starved, or poisoned) under various scenarios. The main utility of ABM in representing the parameters that cause the movement behaviour of individuals and the effects of such behaviours (on the environment, and movement of other individuals) is yet to be examined.

**Figure 1:**
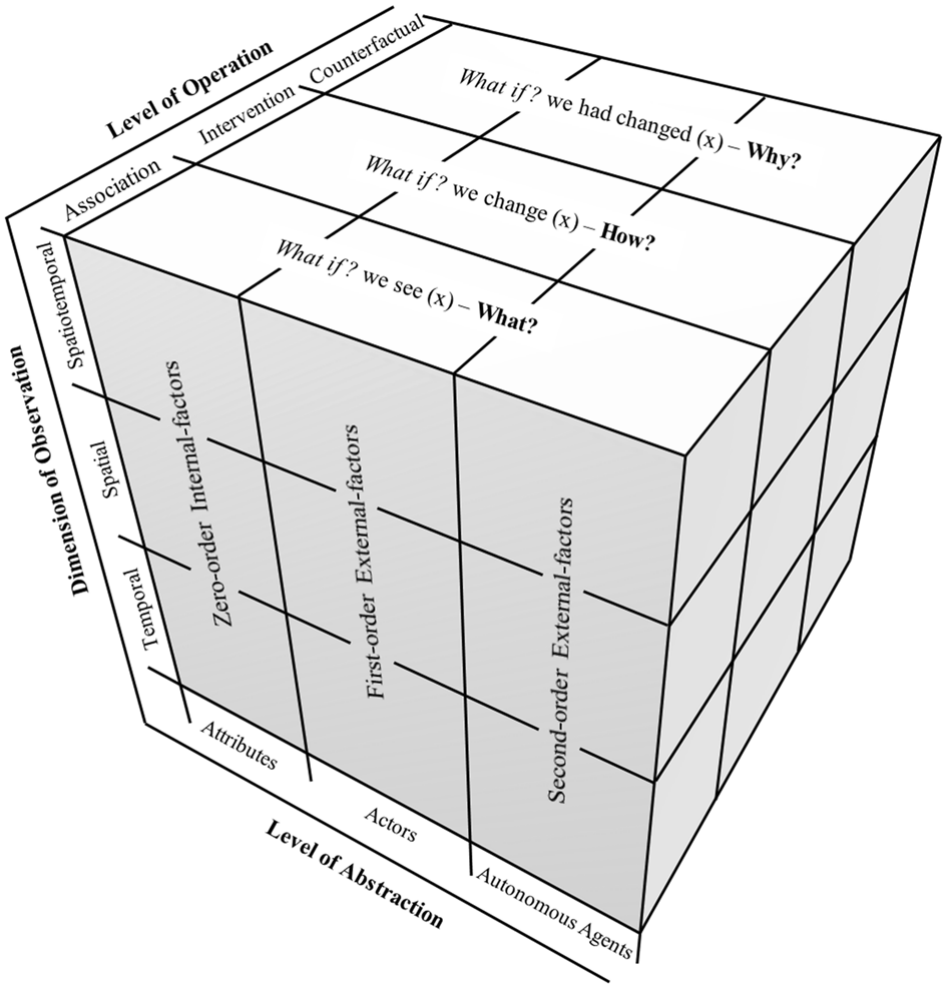
The conceptual representation of intelligent agent queries, given its: state of attributes, interactions with the environmental actors, and relations with other autonomous agents over space and time, and to differing levels of causal analysis, from^46^.

In order to extend the applications of ABM in movement studies, Rahimi, Moore and Whigham^46^ have put forward a conceptual framework based on which ABM could potentially become a credible tool for explicating movement behaviours (Fig. 1).

In this conceptual framework, three levels of abstraction have been proposed to frame an agent-structure for representing movement processes: ‘attribute,’ ‘actor,’ and ‘autonomous agent’. These, in combination with three temporal, spatial, and spatiotemporal general forms of observations, distinguish nine (3 × 3) representation typologies of movement data within the agent framework. Also, to ground this agent-structure, they have abstractly reported an agent-based movement implementation. This paper is the full account of the agent-based movement simulation summarised in Rahimi, Moore and Whigham^46^ that aimed to ground the agent structure proposed in their paper. The current paper attempts to design, implement, and test a replicable model development guideline through which a movement model can acquire explanatory status. The emergent outcomes of the ABMs are often difficult to make sense of. Also, very few statistical tests have been specifically designed for verification and validation of ABMs, particularly for movement models. This paper tries to offer a guideline for dealing with these limitations. Therefore, readers must read the current paper as a contribution to the development of ABM rather than an attempt to model a specific movement phenomenon. In other words, it is specifically an attempt to expand the utility of ABM from mere representation of the movement behaviours to exploring their underlying system of cause and effect.

### Defining a constrained movement context for testing ABM

Movement studies are vast, ranging from humans’ daily commute to animals’ movement in many different forms. A sensible start for testing the explanatory potential of ABM in movement analysis is team sport games during which a group of individuals act in a structured way over a constrained, and a well-defined, spatiotemporal environment^47^. Most team sports tend to be dynamic with multiple players continuously moving and competing against each other, effectively representing autonomous individuals, making decisions within a complex system. The spatial objects, temporal events, time durations and intervals, the agents and their intentions, their associations, and the rules of the game are in most cases well-articulated and understood.

Professional sports analysts are increasingly showing interest in looking beyond match statistics and average individual performances to model movements of players^48^. Among different sports, the motion of football players is perhaps one of the most intensively observed and thoroughly discussed processes that makes explaining and understanding it an interesting and possible research endeavour^49,50^. The majority of conventional applications in football analysis model the probability distribution of events (e.g., ‘expected goals’) over the field^51,52^. Many spatially-concentrated studies, on the other hand, have brought insights into collective movement behaviour of teams by summarising them into visual-analytics products^53–55^. Similar to many other fields, data mining approaches are also prominent, in analysing teams’ tactics and strategies by extracting the sequences of actions and movements^56–62^.

Despite its different application, sport movement analysis is not unfamiliar to movement analysis, and consequently to GIScience^47,63–65^. Both are interested in finding the movement behaviour of an individual (or a group of them), associating such behaviours to their intrinsic characteristics, the contextual environment, and the existence of other individual players. Both fields share the need to understand movement decision-making mechanisms, to explain the spatiotemporal events that underlie their occurrences, and to predict changes in such decisions in response to a change in their internal states or the state of the environment.

Although impressive, none of these methods has fully satisfied the community as they mainly deliver spatial information about *where* and with *how much chance* an event usually happens, but nearly no explanation of *how* and *why* it has happened. This is an overriding problem at the very centre of application studies across spatial science, not just in team sport analysis. Like many other geographic processes, explaining movement behaviours in a game requires a full understanding of a network of inter-dependent causal parameters governing players’ decision-making mechanism. ABM facilitates analysts to explicitly put together various components into a model to regenerate movement behaviours (within a degree of accuracy) and deepen their understanding of such emergent phenomena. Considering all above discussed limitations and requirements, making explicit the complex aspect of a football game would be an ideal, and challenging, case study to evaluate the credibility of ABMs.

### Paper overview

This paper is structured as follows: ‘An agent-based movement simulation’ constitutes the main body of the study that summarizes the development of a spatial agent-based movement model of football players. It starts with an account of three typical required elements of simulation models within ABMs. The target movement process is introduced and described in ‘The problem entity and its conceptualization’ sub-section. ‘Simulation model specification and the computerised model’ is a detailed description of an agent-based football simulation. A mandatory step in ABM is the verification and validation process, where the reliability of the model gets tested: ‘Simulation model Verification and Validation’ section. The paper concludes through a discussion of how ABMs can contribute to our understanding of movement, and ends with an outlook of future studies in movement modelling.

### An agent-based movement simulation

ABM belongs to a larger family of simulation tools, where the hypothetical mechanisms underlying a complex real-world phenomenon are explored within a simulated world. Simulation models in general include three main elements^66^: ‘Problem Entity,’ ‘Conceptual Model,’ and ‘Computerized Model.’ A simulating practice begins with a conceptual model, where the problem entity (e.g., its elements, their attributes, and the underlying rules of their interactions) is mathematically, logically, or verbally represented. Such a conceptual representation is formalised in a simulation model specification—a detailed description of the model and its programming process—to be implemented in a computerised model^32^. Agent-based simulation models, in particular, require an explicit clarification of agent architecture, and conceptualization of the entities that represent the environmental and autonomous entities; their instances, structures, attributes, actions, and interaction rules.

### The problem entity and its conceptualization

#### The problem entity

Here, the problem entity is the decision-making mechanism underlying players’ movement in a football match. Briefly, football is a sport between two teams that compete to score goals − to manoeuvre the ball into the opponent’s goal. Spatially, the game takes place on a pitch that is generally 105 m long (from goal to goal) and 68 m wide. Temporally, a game is played over 90 min split into two periods of 45 min (see^67^ for an overview). Each team has up to 11 players that consist of one goalkeeper and ten outfielders, playing in different positions or roles. A role is associated with fuzzy boundaries that mark an area (role-box) that a player should mostly stay and move within (players usually leave their role-area only for a brief period and a specific purpose). Each player will also have their individual attributes (e.g., strength and stamina). The roles are assigned to players, based on their skills and playing styles^68^.

Football is a team game, meaning that although the individuals’ capabilities have a significant impact on their performances, their movement is structured also by the team’s strategy. A specific strategy stipulates how a team should manage space, time, and individual actions during a game^50,69^. In this regard, roles collectively create a spatial arrangement or formation^62^, which reflects a team’s overall respective strategy (e.g., 4-5-1 formation implies a very defensive plan). A formation is a strategic concept that together with the pitch’s elements (goals and boundaries) structure a set of spatial rules. Such rules, along with players’ abilities, govern the interactions between players with the ball and with other players during the game that is reflected in various movement patterns.

#### Conceptual model of the problem entity

Conceptually, the simulation is built upon Rahimi, Moore, & Whigham's^46^ model, adapting the agent-structure for the point-based movement modelling. According to this model, in a football match, moving point objects (the ball, and players) could be represented with one of two conceptually different classes of entity: environmental *actors* and *auto-agents*. The motions of actors generally are passively driven by external forces, while auto-agents rather move due to their internal motivations. Examples of the latter are the football players only, entities that often possess a set of attributes, capabilities to perceive and change the environment, and ability to act from internal motivation. The ball however is represented by environmental actors.

Rahimi, Moore and Whigham's^46^ model also distinguishes three set of causes of movement and classify them into Zero-, First-, and Second-order factors. Applying this classification, the first set entails the players’ identities and abilities. The second set of factors are contextual causes that includes the pitch elements (goals and boundaries), zones implied by players’ roles, and the ball. The last set of causal factors involves interactions with other players (e.g., passing the ball to a teammate, duelling with an opponent for ball possession, or running away from opponents to create space).

### Simulation model specification and the computerised model

#### Functions and actions: dynamic mechanisms of the model

The rules of interaction are the dynamic mechanisms of a model^70^, with the football match being very complex, thus challenging to simulate the organisation of its interaction rules. Accordingly, the dynamic mechanism of a football match has to be simplified into a set of basic actions, presented in Table 1 (see^71^ for a detailed list of interactions, i.e. events). As the ball is central to all actions in football^72^, in this table, the initial assumption is that the ball instigates all interactions in the game. Table 1 is effectively the mental model of player agents. It shows that players perceive the environment under four scenarios, instigated from the question *Who possesses the ball?*, then choose from seven different actions. All actions lead to the same final stage to decide where to *Move to*? that is a function of a set of five causal factors: *What are my abilities?*; *Where is the goal?*; *Where is the ball?*; *Where are the others?*; and *Where am I?*

**Table 1:** The players’ perception of the environment and their respective actions.

Perception					Action	
***Who possesses the ball?***	*Opponents*	*Can I get the ball?*	*No*	and (the ball is far away)	Act-1*	***Where to Move To?***
				but (the ball is close)	Act-2*	
	*No one*		*Yes*	but (the 1st and 2nd defenders*^1^ are already on it)		
				and (I am the 1st or the 2nd defender)	Act-3*	
	*Me*	*What can I do with it?*	(there is no opponent player between me and the goal*^2^) or (there is one but my dribbling*^3^ ability is higher than passing)		Act-4*	
			(I will be leaving my role-box) or (there is an opponent player between me and the opponent goal and my passing ability is higher than dribbling)		Act-5*	
			(I am close to the opponent goal and there are less than two opponents between me and the goal) or (I am close to our goal and there is an opponent close to me)		Act-6*	
	*My teammate*		I will not be leaving my role-box		Act-7*	

*Move randomly (Act-1), mark the nearest opponent (Act-2), get possession of the ball (Act-3), carry the ball (Act-4), pass the ball (Act-5), shoot the ball (Act-6), open-up the space (Act-7).

*^1^ The 1st defender refers to the closest defensive player to the ball, while the 2nd defender is the immediate support for the 1st one.

*^2^ Does not include the goalkeeper.

*^3^ Dribbling is manoeuvring the ball while moving in a given direction, avoiding defenders' attempts to intercept the ball.

Also, to reduce complexity, certain assumptions are made, and aspects of the game are ignored. These include:

- Limiting strategies for formations (3-5-2, 4-4-2, or 4-3-3 options only), marking plans (fixed marked opponents or marking the closest player), applying pressure on the ball (two closest players, maximum), and passing policies (no backward passes).
- Only a few basic physiological and technical attributes of players are considered (see Table 2); all mental and psychological states are discounted in the model.
- All other contextual parameters such as the weather, the crowd, playing home or away are assumed to be fixed.
- Not modelling any event that interrupts the match (fouls, offsides, corner kicks, throw-ins; goals are the exception), or substitutions.

**Table 2:** Players’ endogenous attributes (Energy is reduced over time, and a few other attributes fluctuate as a function of energy-level).

ID	Team A											Team B										
	1	2	3	4	5	6	7	8	9	10	11	12	13	14	15	16	17	18	19	20	21	22
Energy	100	100	100	100	100	100	100	100	100	100	100	100	100	100	100	100	100	100	100	100	100	100
Stamina	90	79	76	61	78	81	58	66	84	69	65	88	94	79	79	78	72	68	66	84	83	76
Pace	46	74	65	94	80	40	71	65	84	93	93	55	83	79	78	82	52	75	73	92	97	79
Shooting	88	64	61	72	69	62	70	84	94	94	73	73	75	45	69	45	86	81	88	96	93	86
Agility	10	71	66	84	80	78	90	82	90	99	88	10	85	65	85	79	85	91	91	93	91	84
Teamwork	95	71	69	78	85	79	87	86	83	91	77	95	85	61	80	73	92	89	87	86	89	81

**Figure 2:**
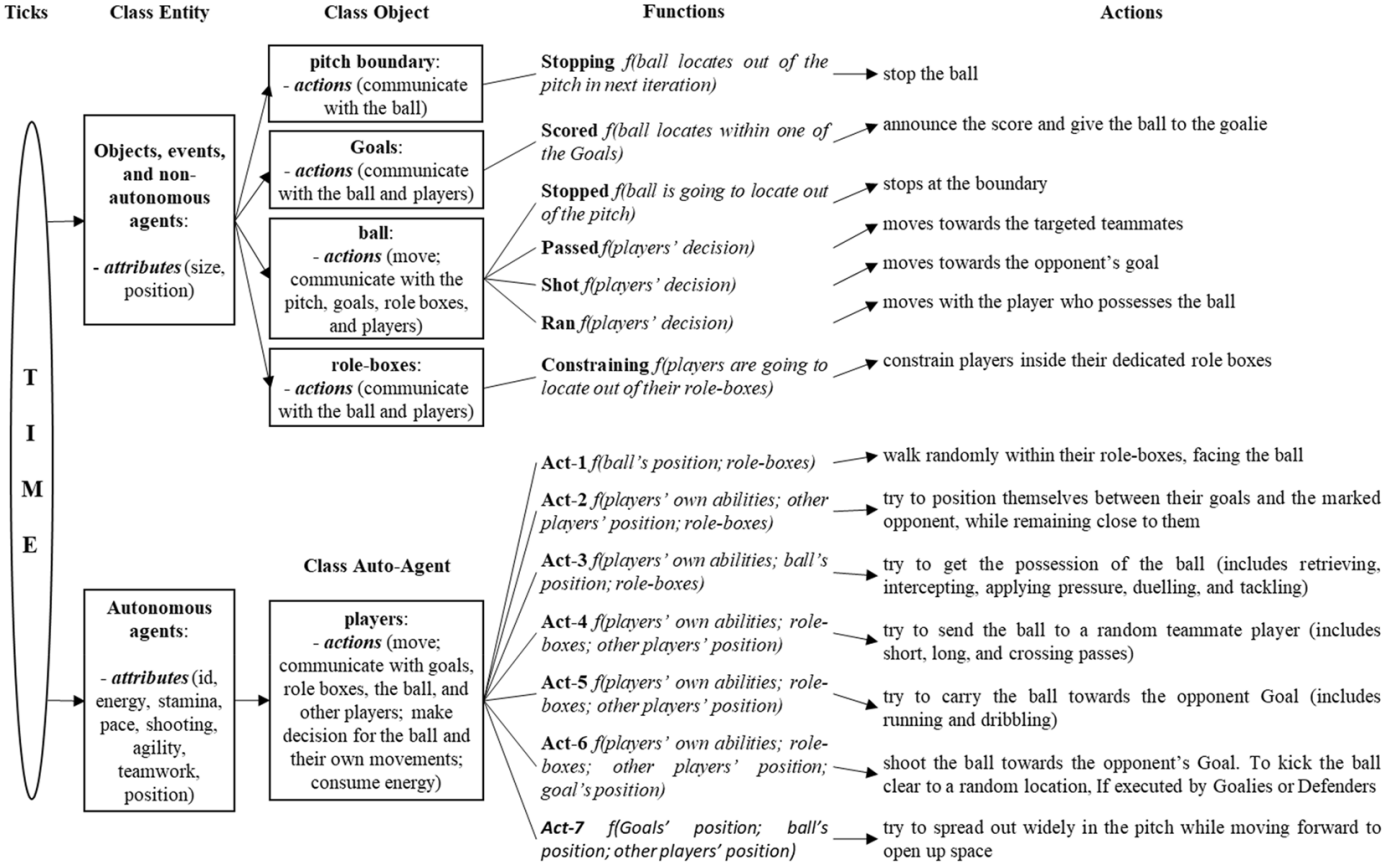
The structure of the model. Adapted with permission from S.C. Ahearn, J.L.D. Smith, A.R. Joshi and J. Ding, Ecological Modelling; Elsevier, 2001.

Drawing the object/agent structure and dynamic mechanisms together, Fig. 2 shows an abstracted structure of the model. According to this structure, players follow slightly different instructions due to their roles. For example, goalkeepers and defenders execute Act-6 (shoot the ball) when an opposing player is pressing them close to their goal (this is rather a defensive act than an action for scoring). Goalkeepers also execute Act-2 (mark an opponent) differently, they try to be at a spot between the ball and their goal when the ball gets close.

#### Agent architecture and modelling environment

An auto-agent theoretically has a brain-inspired mechanism to determine which action it should take regarding its internal and contextual statuses. This is referred to as ‘agent architecture’ that practically controls how the knowledge is represented in the auto-agent and denotes the reasoning mechanism underlying its choices^73^. In the current model, the environment state has assumed to be in one of the ${\varvec{S}}{ }\left\{ {s_{1} ,{ }s_{2} ,{ } \ldots } \right\}$ scenarios, at any given instant, which leads to players’ decision to choose among a set of ${\varvec{A}}{ }\left\{ {a_{1} ,{ }a_{2} ,{ } \ldots } \right\}$ actions. Each player considers a set of causal factors ${\varvec{F}}{ }\left\{ {f_{1} ,{ }f_{2} ,{ } \ldots } \right\}$ to assess $s_{i}$ and to plan $a_{i}$ in more detail (Table 1). Therefore, the players’ movement decision-making process can be simply denoted as a function $m:S \to F \to A$. According to this representation no memory or learning ability are given to these players, thus their decisions are entirely founded on the current situation. This is what Wooldridge^74^ refers to as ‘purely reactive’ architecture, where agents’ decisions are direct stimulus-responses to the current state of the environment. The simplicity and transparency of the players’ decision-making process within purely reactive architecture can help to explicitly interpret their behaviour.

NetLogo has been chosen as the modelling platform to develop the simulation as it simply manifests both Vector and Raster representation of the real world in the form of ‘Turtle’ and ‘Patch’, respectively. NetLogo is an increasingly-used multi-agent modelling environment for computer simulation of complex phenomena^75,76^. It facilitates a visual reconstruction of both contextual actors and auto-agents, and the communication between their instances. These together with its simple ‘if condition, then action’ primitives are well-suited for developing a reactive agent.

#### Model inputs

While the interaction rules are the dynamics of a model, the initial conditions are the history of it^70^. The initialization of our model begins with breeding 22 players for two teams (team A and team B) with *turtle* agents. Each player owns an ID and a set of attributes that are assigned to it as in Table 2. These attributes are either static (e.g., stamina) or dynamic (e.g., energy is systematically reduced over time as a function of stamina). However, pace, shooting, agility, and teamwork are assumed to be static, unless reduced during the game due to energy level.

**Figure 3:**
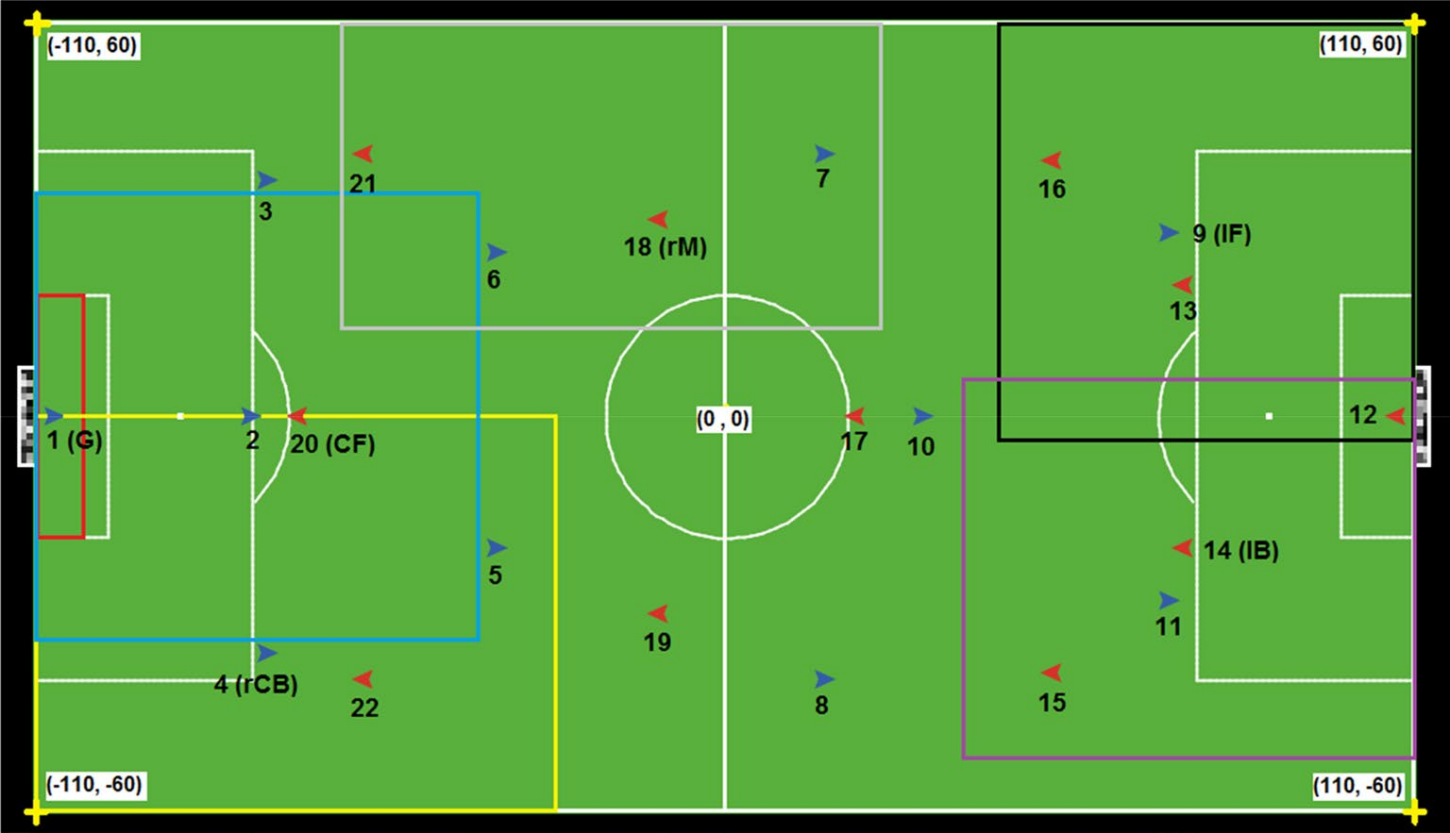
Six examples of the assumed spatial restrictions (role-boxes) for agents. The measurements are relative to a Cartesian coordinate system with the origin point at the middle of the pitch (from^46^). Team A and Team B are represented in blue and red, respectively.

Players are located within a 110-m by 60-m field that is created with 0.5 * 0.5-m *patches* (pixels). Each team is given 3 formation options (3-5-2, 4–4-2, and 4-3-3) that consist of 11 role-boxes. Each role-box is created by a set of patches that communicates with the players and the ball as one agent (environmental actor). In the specific scenario below, we gave Team A (shown in blue) the common formation 3-5-2 and 4-3-3 for team B (shown in red) as presented in Fig. 3. In this figure, red, yellow and black boxes show Goalkeeper (G), right Central Back (rCB), and left Forward (lF) role areas for player 1, 4, and 9. The purple, grey, and blue boxes represent left Back (lB), right Mid-Fielder (rMF), and Central Forward (CF) role domains for player 14, 18, and 20. In the end, a 100 ms time-interval is set to update and record agents’ positions, actions, and attributes during the simulation.

### Simulation model verification and validation

**Figure 4:**
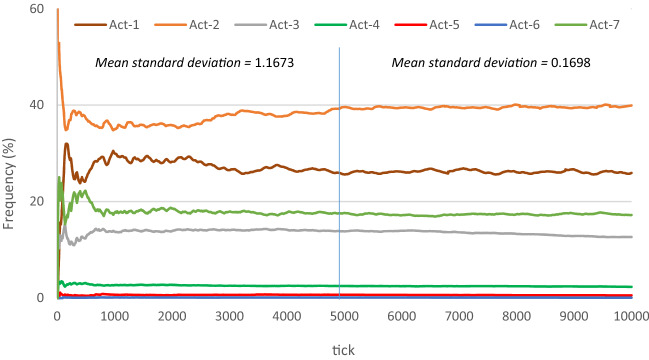
The frequency of executed actions, aggregated from all players in each time steps. The Y axis shows the percentage of each action executed by all players at each time step (X axis). The mean standard deviation of all actions from tick 1–5000, and from 5001 to 10000 are represented in the figure. Actions are abbreviated as follows: move randomly (Act-1), mark the nearest opponent (Act-2), get possession of the ball (Act-3), carry the ball (Act-4), pass the ball (Act-5), shoot the ball (Act-6), open-up the space (Act-7).

A comprehensive verification and validation of all three modelling elements (Problem Entity, Conceptual Model, and Computerized Model) may never be an achievable objective. Conducting small tests and evaluations during modelling to reach a degree of confidence is an accepted and more reasonable alternative^77^. Before conducting these tests, we need to determine how many time intervals (ticks) can represent the model in the verification process. According to an analysis of all actions executed by all players (Fig. 4), 5000 ticks (five hundred simulated seconds − 10 frames per second) can effectively represent the model behaviour.

#### Computerized model verification

**Figure 5:**
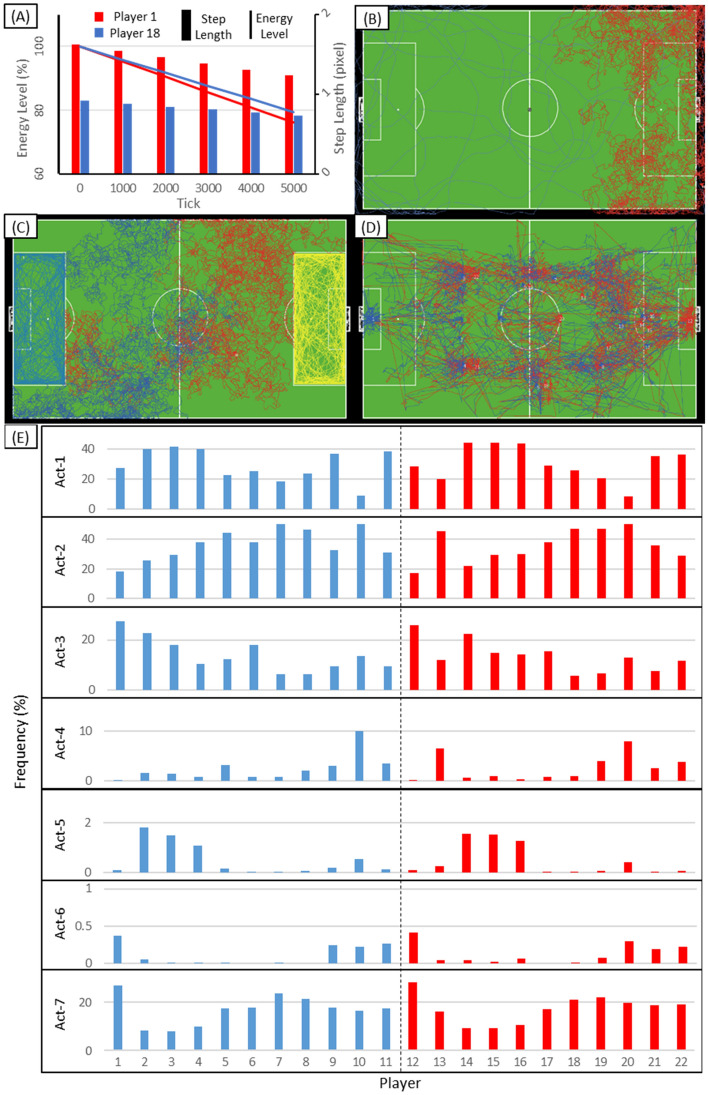
The results of three sets of tests verifying that the players comply with the model specification: (**A**) energy level and step-length (the travelled distance in each time step); (**B**) trajectories of two players, constrained with the zero-order causes; (**C**) trajectories of 4 players, constrained with first-order causes; (**D**) trajectories of 22 players, constrained with all three causal-factors; (**E**) players’ decisions on the executed actions. Note that the Y axis in E is represented in different scales due to the various frequency of the executed actions (e.g., the number of times that players possess the ball, so need to execute Act-4 to 6, is much smaller than other actions). Actions are abbreviated as follows: move randomly (Act-1), mark the nearest opponent (Act-2), get possession of the ball (Act-3), carry the ball (Act-4), pass the ball (Act-5), shoot the ball (Act-6), open-up the space (Act-7).

Three simple statistical and visual tests are conducted to evaluate if agents follow the given decision-making process in Fig. 2. Figure 5 shows graphical summaries of model runs, demonstrating that agents behave as per their parameters:

- Figure 5A shows the energy consumption trend for two agents (the graph) and their average step-length (the bar chart) during the simulation. Note that the maximum permitted step-length and turning-angle is 2 pixels (1 m) and 180 degrees at each tick (100 ms). Also, only two agents were used to facilitate the presentation of results.
- Figure 5B represents the emergent trajectories in that Player 18 (in red) has acted in a more variable pattern, compared to Player 1 (in Blue), due to its higher agility (referring to Table 2).
- Four agents (Player 1 and 10 from Team A, and Player 13 and 18 from Team B) were necessary for the second test to show that players are aware of the spatial constraints (interact with the contextual actors) and move, albeit randomly, within their assumed role-boxes. This is shown in Fig. 5C. Note that Fig. 5C assumes different role-boxes from those presented in Fig. 3. This is to cover the entire pitch and test the players’ awareness of the boundaries. For example, the light-blue and yellow trajectories clearly reveal the respective goalkeepers’ role boxes.
- The emergent space–time behaviour of all 22 players is also summarized in the plotted trajectories in Fig. 5D. The numerical results are backed by this plot, the higher density of trajectories in team B’s half of the pitch verifies slightly more roaming around (Act-1) for defenders in team A. This figure presents the trajectories of 22 players, constrained with all three causal-factors (their own internal states, environmental factors, and other players’ behaviours).
- Figure 5E illustrates players’ decisions on the executed actions (defined in Fig. 2). It appears that midfielders in both teams have decided to watch an opponent player (Act-2) and to create space (Act-7) more than others. This is perhaps because the game has been mostly played around the centre of the pitch.

#### Operational validity

The sensitivity-variability analysis is traditionally a mandatory step in validating model operation^78^. Sensitivity analysis is usually conducted to confirm that the outcomes of the simulation are robust to parameter values, corresponding to the initial assumptions, and to bring insights into the modelled system^79,80^. It is therefore critical to inspect model behaviour under various configurations to understand the potential mechanisms upon which the complex movement patterns emerge. A series of tests are performed: first, to identify model factors that are responsible for the behaviour of players; second, to quantify the effect of these parameters on the model outcomes. It is important to state that the existing statistical methods are not developed specifically for ABMs, thus have no mechanistic focus^81^. Therefore, an interpretation of their results is often required by modellers.

ABMs are often developed based on a collection of assumptions encompassing various parameters, each of which has a broad range of statuses. A further complication is the fact that agent-based movement simulations generate multi-dimensional outcomes, which are extremely challenging for sensitivity analysis. Analysing the whole outcome space corresponding to the variation of all parameters would be impractical due to the computational burden. One way to deal with this kind of problem is to choose a portion of outcomes that can sufficiently portray the true reflection of the model behaviour^82^. Table 3 presents a list of selected outcomes.

**Table 3:** A list of the selected﻿ outcom﻿e space.

Outcomes	**Individual, role-based, team, and aggregated performances**
**Executed actions**	- The frequency of executed actions
**Movement related**	- The distribution of players around the centre of their role-boxes - Players’ movement relative to: The ball’s position The goals Other players (opponents and teammates)
**General statistics**	- Ball possession patterns (sweeping among players and teams)

Regarding to the model setups, a well-known approach is called one-parameter-at-a-time in which a variable is forced to be different, while the context is held fixed in the model, to measure its average effect on the outcomes. This method is widespread due to its simplicity, low computational cost, and transparency for tracing the non-linear cause and effect relationships within complex systems^83^. Modellers can make a list of parameters that are thought to have a greater impact on the model dynamics. The current ABM has a particular property that reduces the eligible parameters, subject to manipulation: most of its key parameters are based on the fixed real values. The number of players, their limitations (i.e. maximum speed and agility), the characteristics of the field, the principal of the game (i.e. interacting with teammates and opponents) are a few examples that cannot be manipulated or permit a limited variation. Table 4 shows a set of parameters selected to represent all three assumed categories of causal-factors.

**Table 4:** A short list of the adjustable model parameters.

**Default setting**	Energy level: players consume energy Formation: team A and B both play with (3-5-2) formation Marking Strategy: both teams have man-to-man marking plan	C1
**2nd configuration**	Energy consumption is changed (players do not consume energy)	C2
**3rd configuration**	Formation is changed: team B with (4-4-3) formation	C3
**4th configuration**	Formation is changed: team A plays with (4-4-3)	C4
**5th configuration**	Marking Strategy is changed: both teams follow zonal marking plan	C5

During the sensitivity-variability analysis modelers must also find the number of replicate runs per parameter setting in whichtheir model behaviour stabilises. This is to determine how many times a model must be run to generate an output that can truly represent the model behaviour. We run a pre-test to examine the distribution of the model outcomes, again in terms of players’ selections of actions. It is recommended to use coefficient of variation, a dimensionless and normalised measure of variance, which is particularly useful when measuring the variance of multiple responses^84^. According to this measure, the model behaviour in the default configuration stabilises at approximately 20 replicate runs.

**Figure 6:**
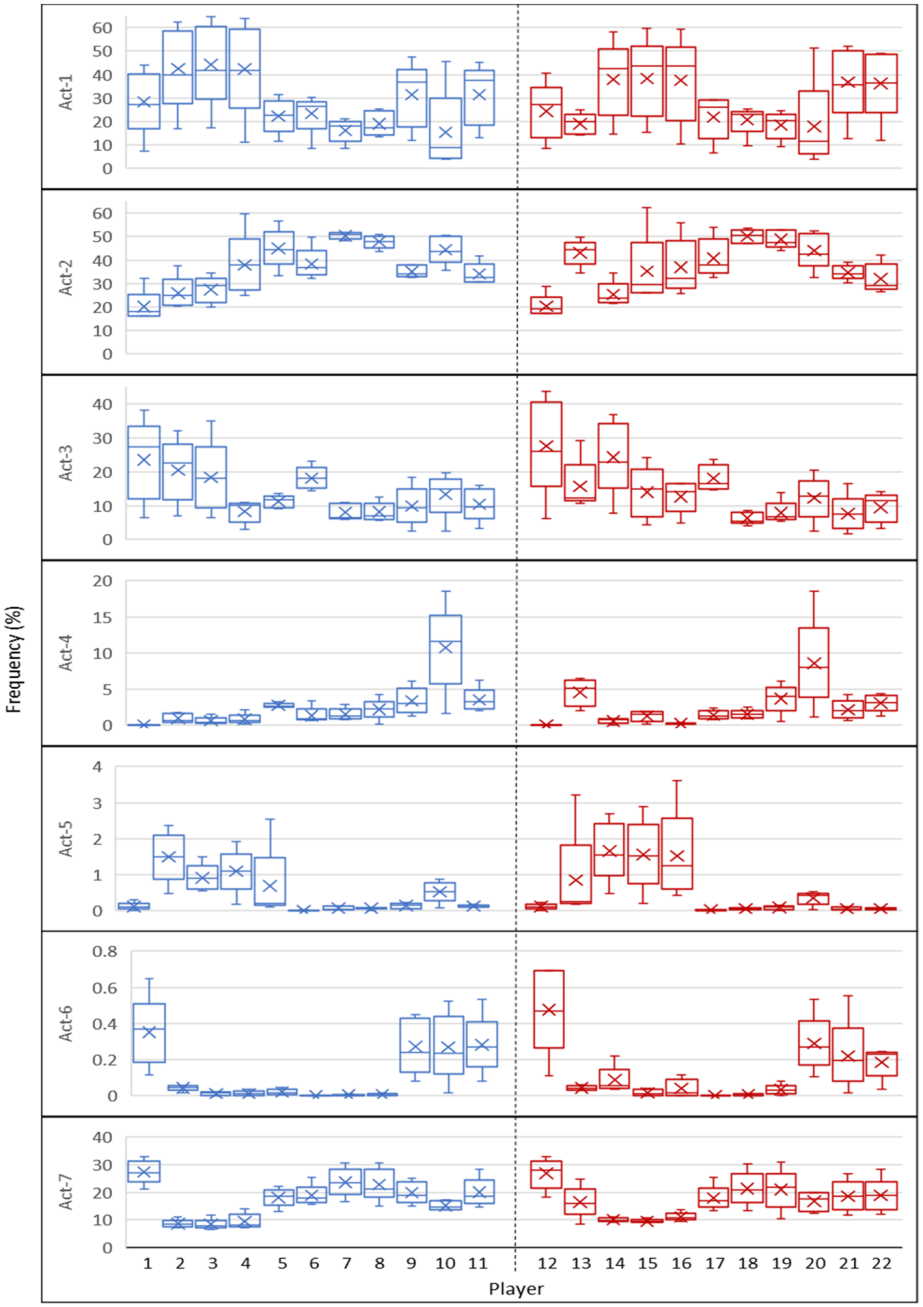
The variation of executed actions over all configurations, at individual-level. Note that the Y axis is represented with different scales due to the various frequency of the executed actions. Act-4 to 6, for example, do not get executed often because they require players to possess the ball. The middle horizontal line and the cross sign, respectively, represent the median and mean of each action across all configurations. Actions are abbreviated as follows: move randomly (Act-1), mark the nearest opponent (Act-2), get possession of the ball (Act-3), carry the ball (Act-4), pass the ball (Act-5), shoot the ball (Act-6), open-up the space (Act-7).

Figure 6 shows the frequency of executed actions over all settings for each player. Regarding Act-1 (random-walk, Table 1), Players 2, 3, 4, 14, 15, and 16 show a higher mean value and variation over different configurations. These players, however, have a relatively smaller mean value, but still a high variation, in Act-2 (watch the opponent) and Act-3 (try to get the ball). Opposite to Act-1, their behaviour on Act-7 (create space) shows a lower mean and variance. Act-1 to 3 are more defensive which may be an explanation for such a variation in the behaviour of these players. This is complemented by more robust behaviour for Players 7, 8, 18, and 19 (they are midfielders in all settings) regarding these acts.

**Figure 7:**
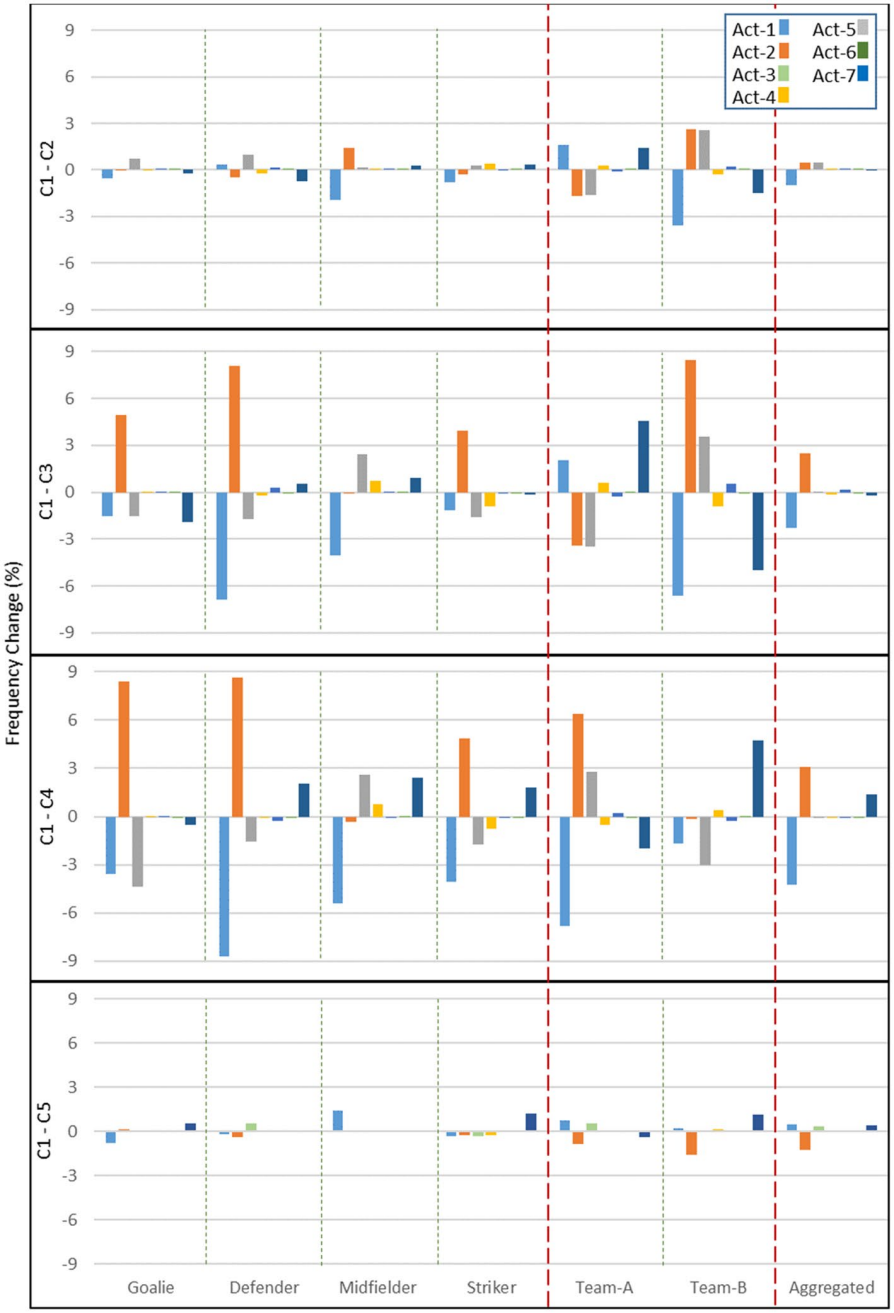
The variation of executed actions over different parameter settings in comparison to the default configuration (C1), at role-, team-, and aggregated-level. Actions are abbreviated as follows: move randomly (Act-1), mark the nearest opponent (Act-2), get possession of the ball (Act-3), carry the ball (Act-4), pass the ball (Act-5), shoot the ball (Act-6), open-up the space (Act-7). The configurations are as follows: default (C1), zero energy consumption (C2), changed formation for Team B (C3), changed formation for Team A (C4), and alternative marking strategies (C5).

A more aggregated reporting (by role, team and overall) is shown in Fig. 7, revealing that defenders and strikers are more sensitive to change, and midfielders more robust to change (this pattern emerges in Fig. 5, too). The role, however, cannot completely explain the emergent patterns, as there are individual differences within roles (e.g., within defenders), pointing to other factors at play. Comparing a player with its teammate and opposing counterpart filters out the impact of its role and to some extent explains the cause of its decisions. For example, given that Players 2 and 13 always play in similar roles convinces us to keep their endogenous abilities accounted for different behaviours in performing Act-4, Act-5, and Act-6 (carry, pass, and shoot the ball, respectively).

**Figure 8:**
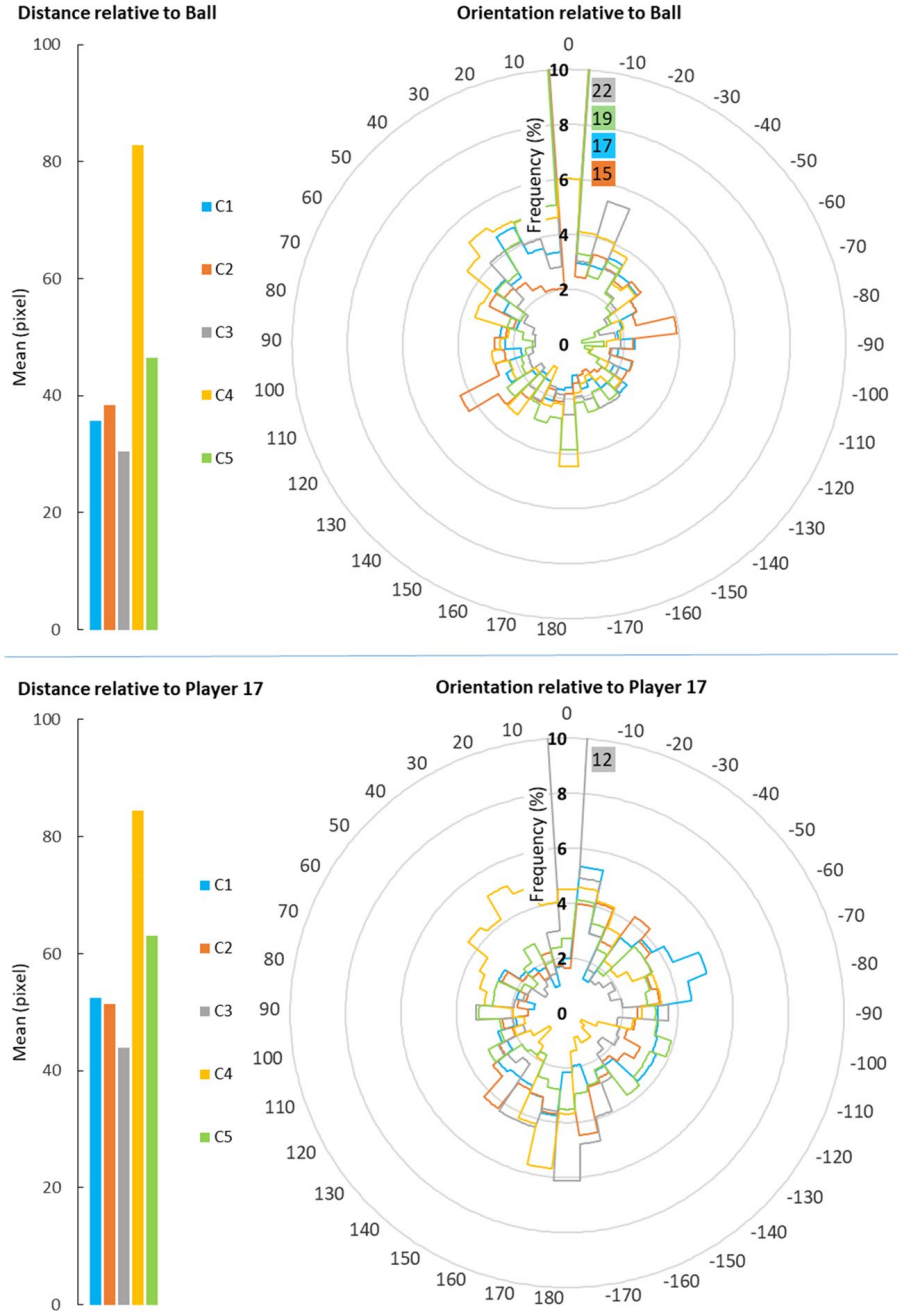
Player 10’s relative distance and orientation towards the ball and Player 17. The relative orientation is represented in 36 classes, 10 degrees in each class. For example, from -5 to -15 is considered in one class (-10), which means Player 10 needs to on average turn 10 degrees in order to directly look at the target. The minus and plus sign, respectively, indicates the left or right turning direction. To be able to effectively show variation amongst the smaller values, the larger values for the northern orientation in some configurations are represented in their correspondent colour next to the northern arm. In the bottom figure, for example, the printed value 12 represents the relative orientation of Player 10 towards Player 17 for class 0 (the northern orientation) in configuration C3, as that value is beyond the radial extent of the diagram. The configurations are as follows: default (C1), zero energy consumption (C2), changed formation for Team B (C3), changed formation for Team A (C4), and alternative marking strategies (C5).

In addition to players’ choices of actions, their movement behaviour is an important outcome that needs to be considered in the variability analysis. Due to space limitations, the presentation of results is limited to Player 10, which appears to be the most active agent. Figure 8 shows the relative orientation and distance of Player 10 towards the ball and Player 17 (see the Data availability section for more player pairings): the ball is the centre of all decisions; and Player 17 is on average the closest agent to our target player. According to this figure, Player 10’s heading is oriented towards the ball for almost 17% of time in C1 (default configuration). This decreased to just over 15% in C2 (changing the energy consumption rate to zero), and 6% in C4 (changing team’s formation), while showing an increase to 19% and 22% in C5 (manipulating teams’ marking strategies) and C3 (changing team to an alternative formation; see Table 4 for more detailed description of C1 to C5). Referring to the figure, a high correlation between this player’s orientation and distance relative to both targets: the closer they get the more Player 10 turns towards them. Comparing these two figures reveals another interesting relation: although the average distance between Player 10 and 17 is much less than between Player 10 and the ball, its heading has been more oriented towards the ball. Also, the interactions between Player 10 and 17 appears to be quite consistent over various settings. The only exception occurred in C3, where they become each other’s direct opponent leading to more interactions.

**Figure 9:**
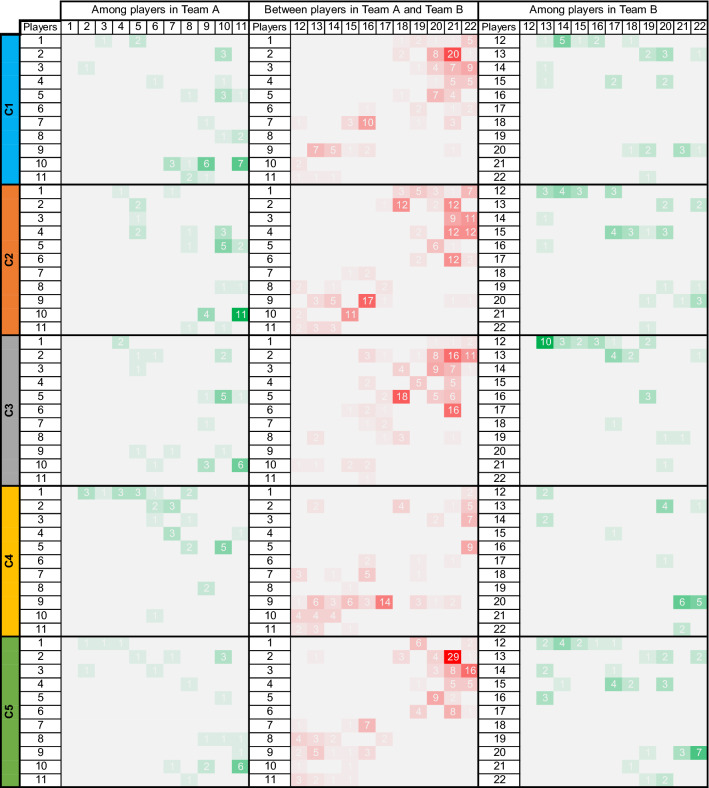
Changing the ball possession among players and between teams. The green colour indicates successful passes among teammates. These values should be read from Player on the row to Player in the column. The red cells show the accumulated number of successful attempts to *get the ball* (interceptions, retrieves, tackles, etc.) that has caused changing the ball possession between teams, without specifying from and to which two involved players. The configurations are as follows: default (C1), zero energy consumption (C2), changed formation for Team B (C3), changed formation for Team A (C4), and alternative marking strategies (C5).

Another ﻿aspect of the model behaviour is the general statistics of the game such as ball possession. Figure 9 illustrates some interesting patterns of changing the ball possession among players and between teams over various model configurations. Team A seems to establish a network to send the ball from the midfielders to strikers then passing it more actively at the front line. However, Team B shows more interactions between defenders and midfielders. This again is interpreted as the effect of the players’ attributes: strikers in team B have on average a higher value of stamina that makes them able to keep performing Act-4 (running the ball, Table 1) instead of passing it throughout the game. Both teams show a change in their ball possession patterns when manipulating their formations: in C4 for Team A, and in C3 for Team B. According to this figure there has been a high tension between defending line of Team A and the strikers in Team B, except in C4 where Team A has put more pressure on Team B.

Figures 5, 6, 7, 8, 9 show that the behaviour of players in general vary more in response to variation of a team’s formation (C3 and C4) than to changing the energy consumption rate (C2). Comparing the outcomes of primary configuration (C1) with the results after manipulating teams’ marking strategies (C5) shows a negligible change in their behaviour. We believe, this is due to the hard boundaries of the role-boxes that dictate playing styles under different formations. Among executed actions, Act-1, Act-2, Act-3, and Act-4, respectively, fluctuate the most in all 5 setups. Manipulating parameters has a higher impact on the behaviour of the defenders, which can only be explained by their roles. Team A’s behaviour is more stable than Team B, except in C4, where its formation is changed. This may be explained with players’ endogenous abilities that on average are higher in Team B. These results are confirmed at the aggregate level. In the end the behaviour of players does not seem to be interrupted under any of the manipulated parameters, which validates the robustness of the model.

## Discussion and conclusion

An (in-depth) understanding and explanation of any phenomenon, including movement, requires inferring its predominant causes. A practical approach is to provide information about possible mechanisms underlying movement decisions: under what circumstances individuals decide where and when to go, and how one could possibly change those decisions. Agent-based simulation models are useful for this task as they force modellers to be explicit in all factors and conditions that cause movement. Such a capability plays a central role in establishing correspondences between theories and observations in GIScience^85^. For researchers however to develop explanatory models^86^: they must ‘start it simple’ (a less realistic movement model can disclose new ideas and hypotheses); they should ‘experiment, not just explore’ (systematic manipulation of parameter space is time consuming, but it leads to a better understanding of the modelled movement); and they need to ‘test robustness’ (check the stability of model behaviour with respect to variation in the parameters related to initial assumptions).

In a practical implementation of such a modelling process, a simplified version of the movement decision-making process of football players is simulated based on an assumption that all interactions in this game are caused by three Zero-, First-, and Second-order factors. Expressed another way, players decide their objectives, then move (or not) to achieve them, after considering: (a) their internal capabilities (energy, pace, stamina, agility, teamwork, and shooting); (b) contextual actors (the constituent elements of the pitch and the ball); and finally (c) other autonomous moving agents (teammate and opponent players). Players are represented abstractly by point vector-agents, as in the current model their position is more relevant than other geometric states (size and shape). A simple reactive agent architecture is implemented in order to explicitly show the chain of causes underlying the behaviour of the players.

The model is then submitted to an accreditation process, including an intensive sensitivity-variability analysis to assess the effects of different configurations of system components on the emergent outcomes. Configuration C1 is the default setup of the model. Players’ energy consumption is altered in Configuration C2 to examine the causal effect of the zero-order on their behaviour. In Configurations C3 and C4 each team’s formation is manipulated to test the effect of the first-order causal factors. Finally, Configuration C5 is designed to analyse the effect of the second-order causes (other players) on the agents’ behaviour. The football model appears to be robust in terms of its basic assumptions. The results also show that the emergent behaviour of players vary more when the manipulations target the condition of the environmental actors (role-boxes and the ball), which suggests that the first-order factors are, in this simulation, the main causes of such behaviours. It is speculated to be caused by the hard-bounded role-boxes. Different results are expected by designing more realistic zones (data-driven, fuzzy boundary, dynamic, or even simply, more circular).

This model seems to achieve its explanatory objectives, in the sense that it generates knowledge about the conditions (dynamic mechanisms and initial inputs) under which a particular behaviour of players emerge. It teaches us that although players behave based on their internal states, their decisions are highly influenced by the formation (strategic plan) of the team. The more restrictive these strategies are, the more impact they have on players’ decisions, and more different movement patterns can be seen. Experimenting with the model may lead to an understanding of the extent to which the team strategies replace the impact of the players’ characteristics on their decisions. This is achievable by placing players in different roles, and by cross analysis of their behaviour.

Backed by the experience gained from this study, and given the existence of a rich theoretical background, it is safe to argue that movement analysts are currently capable of developing ABMs to produce or examine causally-relevant evidence. For an ABM to achieve this potential, modellers need to follow a more rigorous process of conceptualization, clarification, verification and validation. According to Casini and Manzo^87^, a credible ABM should be founded on (a) pre-existing theories around the target phenomena, it must directly use the empirical information as its low-level infrastructure (e.g., agents’ attributes, behavioural rules, geographic locations), its macro-level outcomes must be systematically confronted with empirical patterns, and finally it must be submitted to a series of ‘theoretical explorations’ (sensitivity, variability, dispersion, and model analysis).

A large portion of this paper has been dedicated to the ‘theoretical exploration’ of the model. It seems reasonable to claim that our model in a sense meets the 'theoretical realism,’ and ‘empirical calibration’ conditions. ‘Empirical validation,’ however, is omitted in this paper and potentially could be dealt with in a separate study. Data validity is a mandatory, and computationally costly, step to ensure the external validity of the relevant agent-based movement model. One must have a robust foundation to believe that the differences between the simulation and the target movement behaviour do not create an error when transferring the results from one to another.

The necessity of an intensive empirical calibration and validation, however, poses a dilemma in simulation modelling in general, and movement ABMs in particular. Models indeed are developed to perform abstract thought experiments exploring plausible mechanisms that may underlie observed phenomena. Making them more realistic unavoidably adds complexity that undermines their usefulness as an accessible explanatory tool^86^. Likewise, the enthusiasm for ABM initially arises from a gap in our understanding of a complex system of interest, and lack of sufficient observations related to them. An autonomous decision on movement may involve subjective choices, complex psychology, irrational behaviour, covert intentions, and biased perception of the environment, which are often extremely hard to quantify, observe, and validate. Even if one successfully develops an empirically calibrated and validated movement model, an easy explanation of its behaviour would not be the stimulus, especially if we can no longer understand how the model generates the given result. When the model gets as complex as the modelled entity, sophisticated statistical methods are required to interpret its output that conversely erodes the initial advantages of ABM.

Modellers must be aware of this dilemma and choose the right amount of detail while maintaining an acceptable level of accuracy^88^. The complexity and utility of an ABM should be decided and measured based on its primary purpose: whether it states how reality could or would be under certain spatiotemporal conditions or it intends to reproduce an actual system^89^. The former are explanatory models that emphasise the specific aspects of a system, hoping that such laboratory explorations will lead to empirically relevant understandings^90^. These are generally built for theory-development purposes. While the quest to reproduce an actual system includes predictive ABMs that are commonly used for evaluation of empirically gained knowledge, extrapolation of patterns, and prediction of future states.

The simulation reported here is a ‘data-poor’ model of a complex movement process. It is an effort to adapt explanatory and causal-inference thinking within the movement analysis field: an implementation of the framework proposed by^46^ for agent-based representations of movement phenomena. The next practical step towards a causal movement model would be a data-driven agent architecture where the modellers’ initial assumptions, agents’ behaviours, and the outcomes are calibrated, taught, and validated based on movement observations. To test the validity of such a comprehensive architecture, however, we initially require a movement process of which we know in advance its underlying causal mechanism. Since with empirical data we often do not know the underlying cause of the low-level actions (i.e. we might know overall objectives such as “score a goal”, but we don’t know why an individual moved at any point in time), using empirical data to demonstrate the inference of causal rules or interactions may not be feasible. This is in fact another objective of the current modelling practice. The generated movement data from the current model can be used to test the applications of a data-driven agent architecture in dealing with the complexity involved in movement behaviours.

But we propose a different approach from existing applications, where the data-driven information is often limited to a few low-level inputs (e.g., the number of agents). Following the conceptual framework^46^, we suggest that ABMs should be developed based on highly abstracted causal knowledge. The behaviour of more cognitively sophisticated agents, equipped with a graphical causal model, could be empirically calibrated and validated until a degree of confidence is reached. Once achieved, a model of this nature could be treated as a replica of its real-world counterparts to generate outcomes under different assumptions and explore counterfactual possibilities. Such a trustworthy device would facilitate the researcher to intervene and investigate various aspects of a phenomenon that could become extremely useful in all movement analysis application fields. It would also permit movement analysts to simulate their causal claims to see if the dynamics triggered by the model can generate the observed movement patterns. In a bigger picture, it is an opportunity to establish correspondences between theories and observations in GIScience. Developing such an integrated architecture, therefore, can rightly be identified as the next key challenge for agent-based movement modelling.

## Data Availability

More data and figures on verification and validation of the model are available online at https://figshare.com/s/8387c8fe639a0d31d43b.
